# Exposure of Reproductive-Aged Women to Multiple Metals and Its Associations with Unexplained Recurrent Miscarriage

**DOI:** 10.3390/toxics11100830

**Published:** 2023-09-30

**Authors:** Yingying Zhang, Xi Yan, Jianhua Tan, Jifan Tan, Chunsheng Liu, Pan Yang, Yanping Xian, Qiong Wang

**Affiliations:** 1 Reproductive Medicine Center, The First Affiliated Hospital of Sun Yat-sen University, Guangzhou 510632, China; 2 Guangzhou Quality Supervision and Testing Institute, Guangzhou 510632, China; 3 Guangzhou Institute of Food Inspection, Guangzhou 510632, China; 4 Department of Public Health and Preventive Medicine, Jinan University, Guangzhou 510632, China

**Keywords:** metals, miscarriage, lead, calcium, selenium

## Abstract

Exposure to heavy metals exerts toxic effects on female reproduction and embryo development. This study examined the exposure of patients with unexplained recurrent miscarriage (uRM) to multiple metals and the correlations among exposures to different metals. A total of 275 participants were enrolled, including 43 healthy women without previous miscarriage (the control group) and 232 uRM women (the case group); among these uRM women, 159 had two miscarriages (2M), 42 had three miscarriages (3M) and 31 had four or more miscarriages (≥4M). A total of 22 elements were measured in serum samples via inductively coupled plasma–mass spectrometry. The levels of calcium (104.37 mg/L vs. 92.65/93.02/92.61/92.47 mg/L) and selenium (131.85 µg/L vs. 117.80/118.04/115.88/124.35 µg/L) were higher in the controls than in the total uRM group and the 2M, 3M and ≥4M subgroups. The level of vanadium was significantly lower in the controls than in the total uRM group (0.15 µg/L vs. 0.23 µg/L), and the level of lead was lower in the controls than that in the total uRM group and the 2M, 3M and ≥4M subgroups (0.01 µg/L vs. 0.28/0.18/0.63/0.34 µg/L). After adjusting for age, body mass index and education level, calcium and selenium exposure were consistently negatively associated with miscarriage, while lead exposure was positively associated with miscarriage. In addition, the correlations among exposures to different metals slightly differed between the control and uRM groups. Therefore, changes in some metal elements in the blood might be related to the risk of uRM.

## 1. Introduction

Recurrent miscarriage (RM) is a devastating experience that is defined as two or more clinical pregnancy losses [1]. The prevalence of RM has increased, as observed in a 10-year cohort study [2]. A recent study reported that the average prevalence of RM in women of reproductive age was 2.6% worldwide [3]. However, approximately half of RM cases cannot be explained by known factors such as parental chromosome abnormalities, uterine malformations, infections or endocrine and autoimmune disorders [4]. Identifying the potential risks for unexplained RM (uRM) is critical for developing preventative measures and therapeutic treatments for individuals and in the healthcare system.

Exposure to environmental pollutants has garnered considerable attention due to their non-negligible role in the occurrence of miscarriage [3,5,6]. Among the known pollutants, the impact of metal exposure on miscarriage has been investigated for decades. Heavy metals can cross the placenta and accumulate in the fetus [7]. Mercury, cadmium and lead are well-known toxicants that increase the risk of miscarriage [8]. As reported in a published study in Beijing, the tolerable weekly intake values for cadmium and lead were 3.4 and 11.1 μg/kg bw [9]. In addition to exposure to heavy metals, trace elements also play an important role. The levels of the trace elements selenium, zinc and manganese were lower in pregnant women, and manganese levels were positively associated with the risk of maternal and fetal complications [10]. The lowest-observed-adverse-effect levels (LOAELs) for selenium, zinc and manganese are 0.023, 0.91 and 0.14 mg/kg/day, respectively [11,12]. In addition, studies have found that multiple trace elements, including magnesium, antimony, strontium, tin and bismuth, have predictive value for spontaneous miscarriage [13].

However, few studies have examined the associations between uRM and exposure to multiple metals. Most of the studies had small sample sizes and investigated exposure to a single metal in the RM population. Moreover, the conclusions about the impact of copper, lead and manganese exposure on the risk of RM are not consistent [14,15,16,17,18]. This variability is partially due to the heterogeneity of sample types (blood, placenta, hair, etc.), the timing of sample collection (when women were pregnant or not) and the RM population examined (uRM or RM). A study that recruited the largest number of women with uRM was published in 2012 [19]. This study included 90 patients with previous miscarriages (27 with one miscarriage, 23 with two miscarriages and 40 with three or more miscarriages) and investigated only exposure to cadmium. The results showed significantly higher levels of cadmium in women with previous miscarriage than in women without previous miscarriage [19]. Furthermore, no studies have examined the exposure of women with uRM to trace elements such as silver, barium, beryllium, molybdenum, titanium and vanadium or the correlations among metal exposure in women with uRM.

Considering the limited and contradictory evidence about the role of metal exposure in women with uRM, we conducted a case–control study to explore the exposure of women with uRM to multiple metals. We also examined subgroups that varied in the number of exposures and the correlations among exposures to multiple metals.

## 2. Materials and Methods

### 2.1. Study Population

This case–control study was conducted at the First Affiliated Hospital of Sun Yat-sen University from September 2018 to July 2019. All participants lived in the region of Guangdong province. Almost all the women with uRM experienced their last miscarriage within two years at the time of recruitment. They underwent blood tests, and those with known genetic abnormalities; uterine structural abnormalities; or immune, endocrine or coagulation disorders were excluded. The controls were healthy women without previous pregnancy loss who went on to have live births. Women aged below 20 years or over 40 years and those with previous operations or medical treatment related to the ovaries, polycystic ovary syndrome, irregular menstrual cycles, endometriosis, adenomyosis, abnormalities of the uterine cavity or endocrine and immune disorders were excluded. Finally, 43 healthy women without previous miscarriages (the control group) and 232 women with uRM (the total uRM group), including 159 with two miscarriages (the 2M subgroup), 42 with three miscarriages (the 3M subgroup), and 31 with four or more miscarriages (the ≥4M subgroup), were included in this study. All participants provided information on their age, body mass index (BMI), educational level and smoking status. None of the participants were smokers. This study was approved by the ethics committee of our hospital (Approval number: [2018] 202), and informed consent was obtained from the participants prior to conducting the study.

### 2.2. Sample Collection

Blood samples were collected at the first clinic visit. None of the subjects were pregnant at the time of sample collection. All samples from women with miscarriage were taken post miscarriage, samples from the control group were taken prior to the next pregnancy. Serum was obtained via the centrifugation of whole blood samples at 4 °C and 3000 rpm for 10 min within one hour of collection. All serum samples were frozen at −80 °C until element measurement.

### 2.3. Measurement of Metals

The serum concentrations of the following 22 elements were measured via inductively coupled plasma–mass spectrometry (ICP–MS): beryllium (Be), sodium (Na), magnesium (Mg), potassium (K), calcium (Ca), titanium (Ti), vanadium (V), chromium (Cr), manganese (Mn), iron (Fe), cobalt (Co), nickel (Ni), copper (Cu), zinc (Zn), molybdenum (Mo), silver (Ag), cadmium (Cd), antimony (Sb), barium (Ba), thallium (Tl), lead (Pb) and selenium (Se). In brief, 500 μL of serum was transferred to 15 mL centrifuge tubes, each containing 500 μL of interior label (10 μg/L; AccuStandard, New Haven, CT, USA). Thereafter, 1 mL of 65% nitric acid (Fisher, Waltham, MA, USA) was added, and the solutions were mixed. The tubes were placed at 100 °C for two hours until the solution turned clear. Then, the solution was placed at room temperature and adjusted to a volume of 10 mL using purified water (Milli-Q IQ7000, Merck, Darmstadt, Germany). Each batch (20 samples) contained a standard (SeronormTM Trace Elements) and one blank sample (purified water). Samples were tested in a random order, and experimenters were blinded to the groups during the assessment process. Samples were analyzed using an Agilent 7900 ICP–MS device (Agilent, Santa Clara, CA, USA) with optimized equipment parameters. The recoveries were between 85% and 112% for all elements in the blank sample and matrix. The ICP–MS device was calibrated with a minimum of six standards for each element. A calibration curve with an R2 value of ≥0.995 was considered acceptable. The limits of detection (LODs) of the measured elements were calculated as three times the standard deviation of blank controls (ten replicates). LOD/√2 was used for concentrations below the LOD value. The equation and values of R2, range, LOD, %<LOD, geometric mean, and the first and third quartile for each element are shown in Supplementary Table S1. Most subjects had serum concentrations of beryllium, titanium and cadmium lower than the LOD. Therefore, these three elements were not included in further statistical analysis.

### 2.4. Statistical Analysis

The normality of the distributions of baseline characteristics in the study population was assessed. The Shapiro–Wilk method was used for the normality test. Continuous variables are expressed as the mean with standard deviation (SD) or median with the first quartile (Q1) and the third quartile (Q3) and categorical variables are shown as percentages (%). Group differences between two groups were examined using the Student *t* test, Mann–Whitney U test or chi-square test, depending on the data type. The Kruskal–Wallis H test and one-way ANOVA test were used for comparison among multiple groups based on the distribution of data; Bonferroni correction was used for post hoc testing. Spearman correlation analysis was applied to evaluate the relationships among different metals. Concentrations of metals were log-transformed before logistic regression, except for sodium and zinc. Crude and adjusted single-metal logistic regression models were applied to calculate the odds ratio (OR) and 95% confidence interval (95% CI). Age, BMI and education level were included as covariates in the adjusted logistic regression model. In addition, gWQS (generalized weighted quantile sum) regression was also conducted using the “gWQS” package in R (version 4.0.5). Given that some of the examined elements did not have clear prior associations with miscarriage, both positive and negative models were applied. Statistical analyses were conducted using IBM SPSS version 26. *p* values of less than 0.05 were considered statistically significant.

## 3. Results

### 3.1. Characteristics of the Study Participants

The demographic characteristics of women with uRM and healthy controls in this study are presented in Table 1. Significant differences between the controls and women with miscarriages (total uRM/2M/3M/≥4M) were found in age (31.00 years vs. 32.00/31.86/32.17/33.06 years) and education level. No significant differences were observed regarding BMI (21.72 kg/m^2^ vs. 21.31/21.26/21.32/22.25 kg/m^2^) or parity. In addition, there were no significant differences in age, BMI or parity among subgroups with different numbers of miscarriages.

### 3.2. Metal Concentration Differences between Controls and Women with uRM

**Figure 1:**
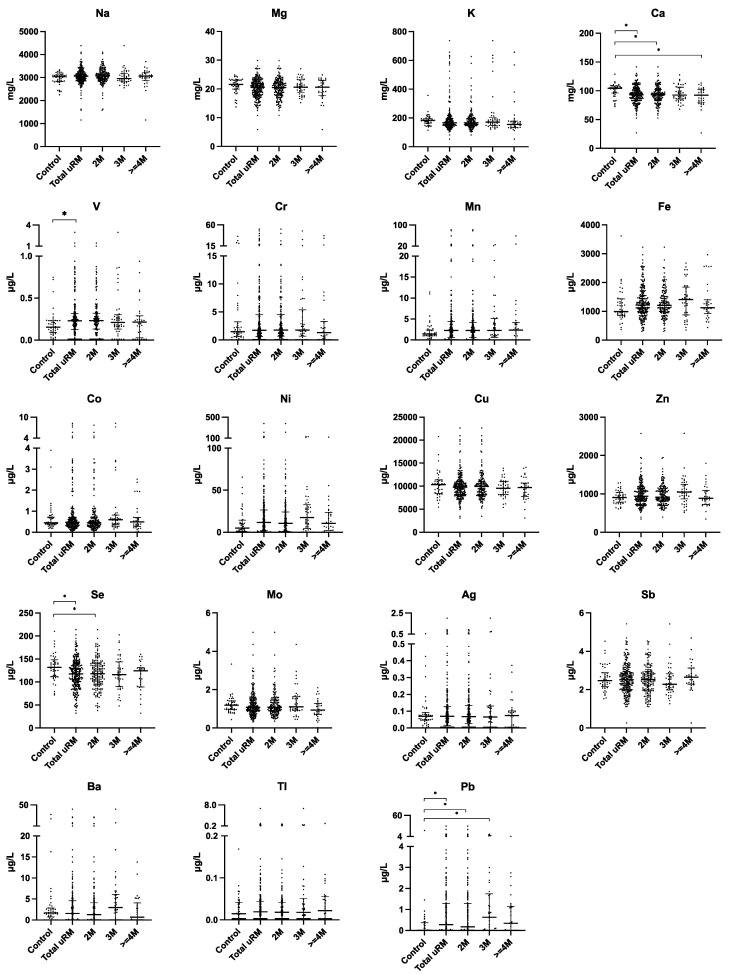
Comparison of serum calcium (Ca), selenium (Se), vanadium (V) and lead (Pb) between control and 2M/3M/≥4M groups. Comparison between control and total uRM group was analyzed via Mann–Whitney U test; multiple analyses among controls and 2M, 3M and ≥4M groups were analyzed via Kruskal–Wallis H test; Bonferroni correction was used for post hoc test. * Means *p*-value < 0.05. Note: 2M, two miscarriages; 3M, three miscarriages; ≥4M, four or more miscarriages.

The level of calcium was significantly higher in the controls than that in the total uRM group (104.37 mg/L vs. 92.65 mg/L). Significant differences were also found between the controls and the 2M subgroup, controls and ≥4M subgroup (104.37 mg/L vs. 93.02, 104.37 mg/L vs. 92.47 mg/L). The adjusted *p* value was less than 0.05 in the comparison between the control and 2M groups (*p* = 0.002), and the control and ≥4M groups (*p* = 0.006). The level of selenium was significantly higher in the controls than that in the total uRM group (131.85 µg/L vs. 117.80 µg/L). Multiple analysis further indicated a significantly higher level of selenium in the controls than that in 2M groups, and a higher but not significant level in the 3M and ≥4M subgroups (131.85 µg/L vs. 118.04 vs. 115.88 vs. 124.35 µg/L). The level of vanadium was significantly lower in the controls than that in the total uRM group (0.15 µg/L vs. 0.23 µg/L). Finally, the level of lead was significantly lower in the control group than that in the total uRM group and the 2M and 3M groups, and lower (but not significantly) than that in ≥4M group (0.01 µg/L vs. 0.28/0.18/0.63/0.34 µg/L) (Table 2 and Figure 1). No significant differences were found when the levels of 19 elements were compared among the 2M, 3M and ≥4M subgroups.

### 3.3. Correlations among Exposures to Multiple Metals

**Figure 2:**
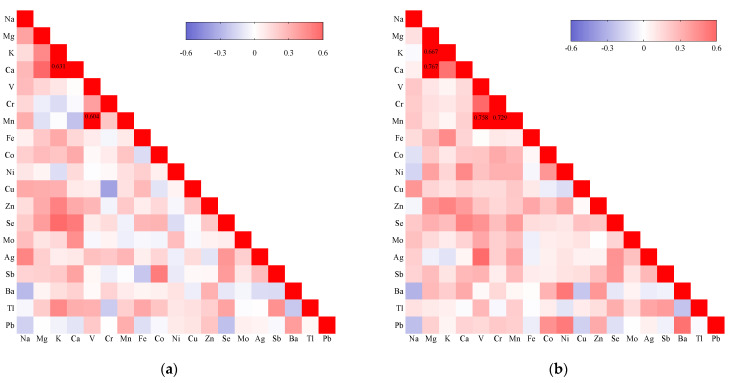
Heatmap of Spearman correlation analysis in control group (**a**) and total unexplained r− current miscarriage (uRM) group (**b**). The correlation heatmaps are used to represent correlation values (r) among multiple metal elements. The colors represent the correlation, with red being more positive, and blue more negative. Only those with correlation values > 0.6 or <−0.6 are marked. Note: Na, sodium; Mg, magnesium; K, potassium; Ca, calcium; V, vanadium; Cr, chromium; Mn, manganese; Fe, iron; Co, cobalt; Ni, nickel; Cu, copper; Zn, zinc; Mo, molybdenum; Ag, silver; Sb, antimony; Ba, barium; Tl, thallium; Pb, lead; Se, selenium.

Most of the metals showed significant but weak correlations (Supplementary Table S2 and Figure 2). The metal correlation patterns slightly differed between the control and uRM groups, with more significant and stronger correlations (r > 0.6) [20] observed in the uRM group. Significant correlations between levels of potassium and magnesium (r = 0.667), calcium and magnesium (r = 0.767), chromium and manganese (r = 0.729), and vanadium and manganese (r = 0.758) were found in the uRM group; in the control group, only the correlations between the levels of potassium and calcium (r = 0.631) and vanadium and manganese (r = 0.604) were significant, and their magnitudes were smaller.

### 3.4. Association between Metal Concentrations and Miscarriage

Logistic regression analysis was conducted to examine the associations between exposures to multiple metals and miscarriages (Table 3). After adjusting for age, BMI and education level, higher levels of calcium and selenium were significantly associated with a lower risk of miscarriage, as revealed by comparisons between the total uRM group and the control group (calcium: OR: 0.012, 95% CI: 0.001–0.188; selenium: OR: 0.141, 95% CI: 0.033–0.597), between the 2M subgroup and the control group (calcium: OR: 0.015, 95% CI: 0.001–0.944; selenium: OR: 0.168, 95% CI: 0.034–0.823), between the 3M subgroup and the control group (calcium: OR: 0.022, 95% CI: 0.919–0.944; selenium: OR: 0.081, 95% CI: 0.012–0.543) and between the ≥4M subgroup and the control group (calcium: OR: 0.003, 95% CI: <0.001–0.189; selenium: OR: 0.076, 95% CI: 0.008–0.751). In addition, higher concentrations of lead were associated with a significantly higher risk of miscarriage, as revealed by comparisons between the total uRM group and the control group (OR: 1.150, 95% CI: 1.003–1.318) and between the 3M subgroup and the control group (OR: 1.258, 95% CI: 1.042–1.519). Higher concentrations of sodium were associated with a significantly higher risk of miscarriage, as revealed by the comparison between the 2M subgroup and the control group (OR: 1.001, 95% CI: 1.000–1.002). Additionally, higher concentrations of zinc (OR: 1.002, 95% CI: 1.000–1.004) and lead (OR: 1.258, 95% CI: 1.042–1.519) and lower concentrations of silver (OR: 0.657, 95% CI: 0.445–0.970) were associated with a significantly higher risk of miscarriage, as revealed by comparisons between the 3M subgroup and the control group.

### 3.5. WQS Regression Analysis of the Association between Blood Metal Concentrations and the Risk of Miscarriage

**Figure 3:**
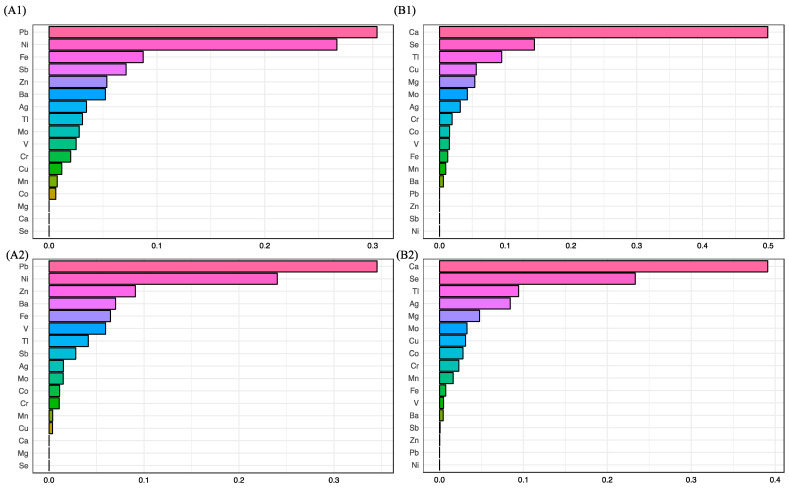
Association between metal levels and uRM status based on weighted quantile sum (WQS) regression analysis. The data modeled without adjustment runs in both positive (**A1**) and negative (**B1**) directions with respect to uRM status. Adjusted models (age, BMI, education level) were also applied in both positive (**A2**) and negative (**B2**) directions. Models with or without adjustments showed similar results, in that both positive and negative associations were found to be associated with uRM. The predominant metal driving positive associations were Pb and Ni, while negative associations was driven primarily by Ca and Se. Note: Na, sodium; Mg, magnesium; K, potassium; Ca, calcium; V, vanadium; Cr, chromium; Mn, manganese; Fe, iron; Co, cobalt; Ni, nickel; Cu, copper; Zn, zinc; Mo, molybdenum; Ag, silver; Sb, antimony; Ba, barium; Tl, thallium; Pb, lead; Se, selenium.

We calculated the WQS index for all metals, where the contribution of each element reflected its relative effect on the risk of miscarriage. The detailed results are presented in Supplementary Tables S3 and S4. Statistically significant associations were found in both the crude model and the adjusted model (*p* < 0.05). The assigned weights for each metal are shown in Figure 3. Based on the mean weight for each metal, the predominant metals driving the positive associations with the risk of miscarriage were lead (crude model: 30.4%; adjusted model: 34.5%) and nickel (crude model: 26.7%; adjusted model: 24%), while the negative associations with the risk of miscarriage were driven primarily by calcium (crude model: 49.9%; adjusted model: 39.1%) and selenium (crude model: 14.4%; adjusted model: 23.3%).

## 4. Discussion

These results indicate that exposure to metals differed in patients with uRM (and uRM patients with different numbers of previous miscarriages) and the controls. Higher levels of lead and lower levels of calcium and selenium were observed in the uRM group, regardless of the number of miscarriages. Higher levels of vanadium were observed in the uRM group compared with the control group. Further logistic and WQS regression indicated that higher lead and nickel levels were associated with a higher risk of miscarriage, while lower calcium and selenium levels were associated with a lower risk of miscarriage. No significant differences in the other metals’ concentrations were found among women in the uRM group, regardless of the number of miscarriages. In addition, the patterns of correlations among metal levels slightly differed between the uRM and control groups, with more significant correlations found in the uRM group.

Calcium is involved in embryo development, decidualization and implantation [21,22,23]. During pregnancy, it is recommended that women consume adequate amounts of nutrients such as calcium, folate, iron and vitamin D [24]. However, previous findings regarding the role of calcium in pregnancy loss are inconsistent. Two earlier studies found no significant difference in calcium levels between patients with miscarriage or threatened miscarriage [25,26], whereas another recent study reported significantly higher calcium levels in women with miscarriage [27]. Regarding women with uRM, previous studies with small sample sizes have found no significant difference in blood calcium concentrations in uRM [16,28]. However, the present study found significantly lower calcium levels in the uRM group. The change in the calcium level is closely related to the level of vitamin D; vitamin D deficiency increases the risk of pregnancy loss [29]. In addition, vitamin D deficiency or insufficiency was associated with immunological dysregulation in women with RM [30]. This indicates that monitoring levels of calcium and vitamin D is important in women with RM. Furthermore, more studies should examine the mechanism of low-level calcium exposure in women with uRM.

Selenium is an important element in the antioxidant family [31] and is critical to normal immune function [32,33]. Diet is the major source of selenium, and the recommended daily dietary intake is 55 µg/L for adult females [34]. Intake of antioxidants may improve the clinical pregnancy rate in subfertile women [35] and benefit the maintenance of early pregnancy [36]. Animal studies have shown that maternal selenium deficiency might induce impairments in progesterone biosynthesis, nutrient transporter expression, placental proliferation and autophagy and promote the immune response in the placenta [37,38]. During normal early pregnancy, serum selenium levels exhibited a decreasing trend, but in women with miscarriage, serum selenium levels exhibited a significant decrease [39]. In this study, the level of selenium was lower in women with uRM than in the controls. The reduced selenium levels in women with uRM in our study are consistent with previous studies on uRM [40,41,42,43] and RM populations [15,16,44]. However, another two studies reported no significant differences in blood or hair selenium concentrations between women with RM and controls [41,45]. Future studies should evaluate the benefit of selenium supplementation for improving pregnancy outcomes.

This is the first study to show that the serum vanadium level was associated with a significantly increased risk of miscarriage. Women with two, three, or four or more miscarriages had a higher level of vanadium than the controls. Compounds containing vanadium, such as vanadate and vanadyl, might be reproductive and developmental toxicants [46]. Exposure to high levels of vanadium may have adverse effects on reproductive function and fetal development in animal experiments [46] and lead to adverse obstetric complications in humans, such as low birthweight [47,48,49], the premature rupture of membranes (PROM) [50] and early-term delivery [49]. However, the reported associations between vanadium levels and spontaneous miscarriage are inconsistent. One study showed significantly higher vanadium levels in serum [13], while two studies showed no significant difference in vanadium levels in placental tissue [51] and blood [52] in women with spontaneous miscarriage compared to healthy pregnant women. Further studies with larger sample sizes are needed to confirm the relationship between vanadium and uRM.

Lead is a well-known heavy metal. Previous studies have reported that high lead concentrations are associated with pregnancy loss [53,54]. Lead exposure could impair fetal bone development and induce neural tube defects, even at relatively low concentrations [55,56]. In addition, lead exposure could reduce the adhesion and regeneration capacity of decidualized cells, compromising embryo implantation and decidualization [57]. The significantly higher concentration of lead in women with uRM compared to the healthy controls found in our study is consistent with previous findings in women with RM [17,18], although another study found no difference in lead levels between women with RM and healthy controls [16]. Previous findings regarding the impact of low-level lead exposure on pregnancy loss are inconsistent [58,59,60,61,62,63,64,65]. The plasma/blood ratio of lead was positively associated with the risk of miscarriage [66]. Therefore, elevated lead levels are believed to be associated with the risk of miscarriage. However, the cutoff value for a higher risk of miscarriage remains unclear, and needs to be investigated in well-designed prospective cohort studies. Furthermore, the mechanism underlying the toxic effects of low-level lead exposure in women with uRM should be explored.

Most correlations among metal levels are positive in spontaneous miscarriage [13]. Positive correlations between metal levels were found in both the control and uRM groups in this study, indicating similar interactions between these metals in healthy individuals and uRM patients. However, more significant correlations among these metals were observed in the uRM group, which might indicate larger synergistic effects of metal exposure in the uRM group.

The lack of significant differences in metal exposure among women with different numbers of miscarriages might be due to our sample size. In addition, samples from women with miscarriage were taken after miscarriage in our study. The association between the risk of miscarriage and metal exposure before and during pregnancy should be explored in future studies based on samples taken at multiple timepoints. In addition, the potential associations among metal levels and the risk of RM (with known causes) were not identified in this study since only women with uRM were included. This study also lacked detailed information on factors such as passive smoking or nonoccupational exposure. Future studies should recruit a larger sample size to confirm the results of our study.

## 5. Conclusions

The present study found higher levels of vanadium and lead and lower levels of calcium and selenium in serum samples from women with uRM compared with healthy controls. Therefore, an assessment of serum metal levels, especially vanadium, lead, calcium and selenium, should be performed in women with uRM to exclude potential risks due to metal exposure. In addition, reducing exposure to vanadium and lead and taking supplemental calcium and selenium might help to decrease the risk of uRM. Future studies with larger sample sizes should be conducted to confirm the results of this study and the mechanism underlying the relationship between metal exposure and uRM.

## Figures and Tables

**Table 1:** General characteristic among groups of different times of previous miscarriage.

Characteristic	Control (N = 43)	Total uRM (N = 232)	2M (N = 159)	3M (N = 42)	≥4M (N = 31)	*p* (Total uRM vs. Control)	*p* (Multiple Comparisons)
Age, year; median (Q1–Q3)	31.00 (26.00–37.00)	32.00 (26.00–38.00)	31.86 (29.00–35.00)	32.17 (30.00–35.00)	33.06 (31.00–35.00)	**0.007**	**0.019 ^a^**
BMI, kg/m^2^; mean (SD)	21.72 (2.47)	21.31 (2.45)	21.26 (2.13)	21.32 (1.79)	22.25 (3.39)	0.313	0.078
Education Level, n (%)						**<0.001**	**<0.001 ^b^**
High school or below	31 (72.1)	91 (39.2)	52 (32.7)	21 (50.0)	18 (58.0)		
College or equivalent	9 (20.9)	128 (55.2)	96 (60.4)	21 (50.0)	11 (35.5)		
Graduate school or above	3 (7.0%)	13 (5.6)	11 (6.9)	0	2 (6.5)		
Parity, n (%)						0.062	0.282
Nulliparous	36 (83.7)	162 (69.8)	110 (69.2)	29 (69.0)	23 (74.2)		
Multiparous	7 (16.3)	70 (30.2)	49 (30.8)	13 (31.0)	8 (25.8)		

Note: Multiple comparisons were conducted among control, 2M, 3M and ≥4M groups. ^a^ means that significant difference was found in age among groups; post hoc analysis showed that adjusted *p* value with <0.05 was found in comparison between control and ≥4M (*p* = 0.016). ^b^ means that significant difference was found in education level among groups. Abbreviations: BMI, body mass index; Q1: quartile 1; Q3: quartile 3. Data presented as mean (SD) were analyzed via *t* test and one-way ANOVA test; data presented as median (Q1–Q3) were analyzed via Mann–Whitney U test or Kruskal–Wallis H test. Significant *p* value was highlighted.

**Table 2:** Profiling of 22 elements in serum of case–control group.

Elements	Control (N = 43)	Total RM (N = 232)	2M (N = 159)	3M (N = 42)	≥4M (N = 31)	*p* (Control vs. Total RM)	*p* (Multiple Comparisons)	*p* (Adjusted)
Na (mg/L)	3035.51 (2832.09–3117.59)	3062.54 (2877.45–3235.85)	3092.00 (2906.18–3248.21)	2957.35 (2834.82–3186.39)	3065.49 (2877.42–3206.23)	0.064	0.072	-
Mg (mg/L)	21.55 (19.95–23.11)	20.57 (18.05–22.49)	20.48 (17.94–22.35)	20.60 (18.80–23.18)	20.60 (17.63–22.98)	0.052	0.228	-
K (mg/L)	183.29 (164.35–194.30)	166.32 (147.80–195.76)	166.55 (148.74–196.86)	171.47 (148.02–201.511)	154.31 (134.62–178.10)	0.059	0.076	-
Ca (mg/L)	104.37 (96.11–108.20)	92.96 (83.52–102.47)	93.02 (84.69–102.24)	92.61 (85.59–106.48)	92.47 (79.04–102.00)	**<0.001**	**0.002**	**0.006** (control vs. ≥4M); **0.002** (control vs. 2M)
V (μg/L)	0.15 (0.09–0.23)	0.23 (0.13–0.32)	0.23 (0.13–0.32)	0.21 (0.13–0.30)	0.21 (0.03–0.29)	**0.013**	0.069	-
Cr (μg/L)	1.49 (0.64–3.22)	1.74 (0.62–4.53)	1.75 (0.68–4.55)	1.79 (0.69–5.39)	1.33 (0.06–3.29)	0.956	0.727	-
Mn (μg/L)	1.43 (0.99–2.39)	2.27 (0.56–4.38)	2.26 (0.58–4.22)	2.29 (0.60–5.10)	2.39 (0.04–4.17)	0.105	0.394	-
Fe (μg/L)	990.315 (2832.09–3117.59)	1222.02 (962.23–1557.26)	1205.75 (981.97–1521.93)	1408.14 (873.45–1846.25)	1123.82 (917.60–1400.30)	0.064	0.198	-
Co (μg/L)	0.44 (0.37–0.72)	0.47 (0.29–0.71)	0.45 (0.28–0.72)	0.60 (0.37–0.82)	0.49 (0.27–0.71)	0.639	0.264	-
Ni (μg/L)	5.02 (0.97–14.27)	11.63 (1.75–26.60)	10.53 (1.02–24.00)	17.46 (4.25–32.78)	10.45 (2.23–23.66)	0.055	0.051	-
Cu (μg/L)	10,321.86 (8396.07–11,374.16)	9798.82 (8159.29–10,976.13)	9933.40 (8180.86–10,978.27)	9546.22 (8183.17–10,996.32)	9711.50 (7801.93–10,677.12)	0.244	0.566	-
Zn (μg/L)	907.08 (782.79–1037.49)	939.02 (807.90–1134.24)	914.36 (807.24–1131.43)	1047.81 (869.05–1246.04)	885.14 (728.82–1090.37)	0.196	0.127	-
Se (μg/L)	131.85 (111.70–148.04)	117.80 (94.10–138.05)	118.04 (95.57–13,922)	115.88 (90.18–143.61)	124.35 (89.00–131.21)	**0.005**	**0.037**	**0.046** (control vs. 2M)
Mo (μg/L)	1.19 (0.96–1.42)	1.07 (0.85–1.46)	1.07 (0.85–1.46)	1.04 (0.92–1.63)	0.94 (0.70–1.28)	0.398	0.121	-
Ag (μg/L)	0.08 (0.05–0.09)	0.07 (0.02–0.13)	0.07 (0.03–0.13)	0.07 (0.00–0.13)	0.07 (0.00–0.10)	0.893	0.945	-
Sb (μg/L)	2.47 (2.15–2.88)	2.51 (2.02–2.94)	2.53 (1.97–2.95)	2.28 (1.98–2.83)	2.65 (2.29–3.13)	0.742	0.346	-
Ba (μg/L)	1.70 (0.74–2.84)	1.56 (0.02–4.61)	1.29 (0.02–4.10)	2.98 (0.02–6.11)	0.69 (0.02–4.05)	0.443	0.092	-
Tl (μg/L)	0.01 (0.00–0.04)	0.02 (0.00–0.04)	0.12 (0.00–0.04)	0.02 (0.00–0.05)	0.02 (0.00–0.06)	0.560	0.784	-
Pb (μg/L)	0.01 (0.01–0.37)	0.28 (0.01–1.28)	0.18 (0.01–1.29)	0.63 (0.01–1.74)	0.34 (0.01–1.15)	**0.002**	**0.010**	**0.045** (control vs. 2M); **0.007** (control vs. 3M)

Note: Data are presented as median (Q1–Q3). Comparisons between two groups were conducted via Mann–Whitney U test; comparison among different RM groups were conducted via Kruskal–Wallis H test. Abbreviations: quartile 1, Q1; quartile 3, Q3; recurrent miscarriage, RM; beryllium, Be; sodium, Na; magnesium, Mg; potassium, K; calcium, Ca; titanium, Ti; vanadium, V; chromium, Cr; manganese, Mn; iron, Fe; cobalt, Co; nickel, Ni; copper, Cu; zinc, Zn; selenium, Se; molybdenum, Mo; silver, Ag; cadmium, Cd; antimony, Sb; barium, Ba; thallium Tl; lead, Pb. Significant *p* value was highlighted.

**Table 3:** Associations between metals and RM.

Elements	Control vs. Total uRM				Control vs. 2M				Control vs. 3M				Control vs. ≥4M			
	Crude		Adjusted		Crude		Adjusted		Crude		Adjusted		Crude		Adjusted	
	OR (95% C.I.)	*p*	OR (95% C.I.)	*p*	OR (95% C.I.)	*p*	OR (95% C.I.)	*p*	OR (95% C.I.)	*p*	OR (95% C.I.)	*p*	OR (95% C.I.)	*p*	OR (95% C.I.)	*p*
Na (mg/L)	1.001 (1.000, 1.002)	0.061	1.001 (1.000, 1.002)	0.058	1.001 (1.000, 1.002)	**0.028**	1.001 (1.000, 1.002)	**0.045**	1.001 (0.999, 1.003)	0.240	1.001 (0.999, 1.003)	0.258	1.000 (0.999, 1.002)	0.623	1.001 (1.000, 1.003)	0.153
Mg (mg/L)	0.167 (0.019, 1.465)	0.106	0.135 (0.013, 1.421)	0.096	0.156 (0.017, 1.429)	0.100	0.126 (0.011, 1.424)	0.094	0.374 (0.016, 8.708)	0.540	0.296 (0.009, 9.938)	0.497	0.097 (0.006, 1.698)	0.110	0.127 (0.005, 3.599)	0.227
K (mg/L)	0.887 (0.346, 2.274)	0.803	0.982 (0.363, 2.655)	0.972	0.669 (0.207, 2.166)	0.503	0.895 (0.248, 3.229)	0.866	2.094 (0.576, 7.604)	0.262	2.142 (0.535, 8.573)	0.282	0.565 (0.129, 2.464)	0.447	0.611 (0.129, 2.903)	0.536
Ca (mg/L)	0.015 (0.001, 0.185)	**0.001**	0.012 (0.001, 0.188)	**0.002**	0.014 (0.001, 0.200)	**0.002**	0.015 (0.001, 0.281)	**0.005**	0.025 (0.001, 0.716)	**0.031**	0.022 (0.001, 0.944)	**0.047**	0.003 (0.000, 0.145)	**0.003**	0.003 (0.000, 0.189)	**0.003**
V (μg/L)	1.110 (0.869, 1.419)	0.403	1.186 (0.901, 1.560)	0.223	1.140 (0.876, 1.482)	0.330	1.346 (0.989, 1.832)	0.059	1.201 (0.829, 1.739)	0.333	1.130 (0.748, 1.707)	0.562	0.954 (0.652, 1.398)	0.810	1.050 (0.694, 1.588)	0.817
Cr (μg/L)	0.941 (0.778, 1.137)	0.526	0.965 (0.786, 1.184)	0.732	0.951 (0.775, 1.167)	0.632	1.002 (0.796, 1.261)	0.987	0.961 (0.751, 1.231)	0.754	0.942 (0.708, 1.252)	0.679	0.844 (0.645, 1.104)	0.216	0.881 (0.652, 1.189)	0.407
Mn (μg/L)	0.990 (0.832, 1.178)	0.906	1.038 (0.856, 1.258)	0.707	1.007 (0.833, 1.217)	0.944	1.109 (0.890, 1.383)	0.356	0.993 (0.769, 1.284)	0.960	0.920 (0.685, 1.235)	0.578	0.889 (0.682, 1.158)	0.385	0.927 (0.699, 1.230)	0.600
Fe (μg/L)	1.779 (0.830, 3.815)	0.139	1.996 (0.882, 4.521)	0.097	1.810 (0.778, 4.211)	0.169	2.010 (0.800, 5.050)	0.138	1.935 (0.756, 4.951)	0.169	1.751 (0.639, 4.798)	0.276	1.716 (0.587, 5.018)	0.324	1.361 (0.444, 4.174)	0.590
Co (μg/L)	0.932 (0.657, 1.324)	0.696	0.877 (0.588, 1.306)	0.518	0.869 (0.595, 1.270)	0.469	0.819 (0.525, 1.278)	0.379	1.450 (0.823, 2.555)	0.199	1.353 (0.721, 2.536)	0.346	0.788 (0.462, 1.347)	0.384	0.717 (0.373, 1.377)	0.317
Ni (μg/L)	1.101 (0.951, 1.275)	0.199	1.092 (0.933, 1.279)	0.273	1.069 (0.917, 1.246)	0.394	1.043 (0.881, 1.235)	0.625	1.296 (1.031, 1.629)	**0.027**	1.283 (0.999, 1.647)	0.051	1.118 (0.884, 1.414)	0.353	1.158 (0.897, 1.495)	0.260
Cu (μg/L)	0.495 (0.140, 1.753)	0.276	0.733 (0.205, 2.919)	0.704	0.628 (0.174, 2.269)	0.477	0.924 (0.229, 3.719)	0.911	0.298 (0.045, 1.088)	0.211	0.415 (0.052, 3.315)	0.407	0.255 (0.043, 1.513)	0.133	0.322 (0.039, 2.656)	0.293
Zn (μg/L)	1.001 (1.000, 1.003)	0.103	1.001 (1.000, 1.003)	0.073	1.001 (1.000, 1.003)	0.157	1.001 (1.000, 1.003)	0.090	1.002 (1.000, 1.005)	**0.017**	1.002 (1.000, 1.004)	**0.047**	1.001 (0.999, 1.003)	0.532	1.001 (0.999, 1.003)	0.371
Se (μg/L)	0.154 (0.041, 0.588)	**0.006**	0.141 (0.033, 0.597)	**0.008**	0.152 (0.037, 0.622)	**0.009**	0.168 (0.034, 0.823)	**0.028**	0.166 (0.032, 0.871)	**0.034**	0.081 (0.012, 0.543)	**0.010**	0.103 (0.015, 0.707)	**0.021**	0.076 (0.008, 0.751)	**0.027**
Mo (μg/L)	0.771 (0.384, 1.548)	0.464	0.789 (0.368, 1.650)	0.514	0.805 (0.358, 1.809)	0.599	1.079 (0.431, 2.699)	0.871	1.337 (0.475, 3.767)	0.582	1.005 (0.320, 3.151)	0.993	0.256 (0.072, 0.905)	**0.034**	0.271 (0.067, 1.098)	0.067
Ag (μg/L)	0.885 (0.698, 1.122)	0.312	0.838 (0.637, 1.101)	0.204	0.904 (0.699, 1.170)	0.443	0.898 (0.656, 1.229)	0.502	0.843 (0.617, 1.154)	0.287	0.657 (0.445, 0.970)	**0.035**	0.746 (0.508, 1.096)	0.136	0.721 (0.463, 1.124)	0.148
Sb (μg/L)	0.669 (0.245, 1.827)	0.433	0.626 (0.230, 1.704)	0.359	0.653 (0.229, 1.861)	0.425	0.630 (0.246, 1.613)	0.336	0.389 (0.077, 1.959)	0.252	0.238 (0.038, 1.497)	0.238	0.947 (0.256, 3.502)	0.935	1.093 (0.252, 4.741)	0.906
Ba (μg/L)	0.873 (0.758, 1.006)	0.060	0.854 (0.735, 0.992)	**0.039**	0.852 (0.74, 0.989)	**0.035**	0.852 (0.723, 1.003)	0.055	0.988 (0.815, 1.197)	0.901	0.924 (0.745, 1.147)	0.473	0.790 (0.638, 0.979)	**0.031**	0.708 (0.543, 0.923)	**0.011**
Tl (μg/L)	1.099 (0.881, 1.372)	0.402	1.129 (0.882, 1.444)	0.335	1.075 (0.846, 1.366)	0.555	1.138 (0.864, 1.500)	0.357	1.174 (0.891, 1.547)	0.254	1.191 (0.866, 1.638)	0.283	1.159 (0.836, 1.607)	0.377	1.097 (0.764, 1.579)	0.620
Pb (μg/L)	1.188 (1.043, 1.354)	**0.009**	1.150 (1.003, 1.318)	**0.045**	1.166 (1.019, 1.333)	**0.025**	1.152 (0.995, 1.333)	0.059	1.298 (1.088, 1.548)	**0.004**	1.258 (1.042, 1.519)	**0.017**	1.215 (1.002, 1.474)	**0.048**	1.123 (0.905, 1.394)	0.293

Note: Binary regression was used to explore the associations between metals and uRM. All metals except Na and Zn were log-transformed before analysis. Covariates of age (continuous), BMI (continuous) and education level (categorical) were used in the adjusted model. Abbreviations: recurrent miscarriage, RM; beryllium, Be; sodium, Na; magnesium, Mg; potassium, K; calcium, Ca; titanium, Ti; vanadium, V; chromium, Cr; manganese, Mn; iron, Fe; cobalt, Co; nickel, Ni; copper, Cu; zinc, Zn; selenium, Se; molybdenum, Mo; silver, Ag; cadmium, Cd; antimony, Sb; barium, Ba; thallium Tl; lead, Pb. Significant *p* value was highlighted.

## Data Availability

Data are available from the corresponding author upon request.

## Supplementary Materials

The following supporting information can be downloaded at: https://www.mdpi.com/article/10.3390/toxics11100830/s1, Supplementary Table S1. Equation, R2, range, level of detection (LOD) and geometric mean of 22 elements; Supplementary Table S2. Spearman correlations among serum concentrations of 19 elements in control group and uRM group; Supplementary Table S3. The association of uRM with the WQS index of metals; Supplementary Table S4. Association between metal composite levels and uRM based on weighted quantile sum (WQS) regression analysis.
