# Supplementary material for: Exposure of Reproductive-Aged Women to Multiple Metals and Its Associations with Unexplained Recurrent Miscarriage
Source: Toxics. 2023 Sep 30;11(10):830. doi: 10.3390/toxics11100830 (PMC10611235; doi:10.3390/toxics11100830)
Supplement: Supplementary file 1 [file toxics-11-00830-s001.zip › toxics-2605558-supplementary.pdf]

Supplemental Materials

Exposures of reproductive-aged women to multiple metals and associations with unexplained recurrent miscarriage

Yingying Zhang, Xi Yan, Jianhua Tan, Jifan Tan, Chunsheng Liu, Pan Yang, Yanping Xian, Qiong Wang

Supplementary Table S1. Equation, R2, range, level of detection (LOD) and geometric of 22 elements.

Elements	Equation	R^2	Range	LOD	% < LOD	Geometric Mean (Q1-Q3)
Be (μg/L)	Y=7.6790E-004*x+2.2534E-005	1	0.1-100	0.023	83.64	0.13 (0.02-0.02)
Na (mg/L)	Y=17.5553*x+1.0366	1	0.1-5.0	0.034	0.00	3049.39 (2872.62-3203.76)
Mg (mg/L)	Y=8.8780*x+0.0184	1	0.1-5.0	0.0038	0.00	20.40 (18.31-22.82)
K (mg/L)	Y=3.8858*x+0.2350	0.9999	0.1-5.0	0.015	0.00	191.93 (148.10-194.77)
Ca (mg/L)	Y=0.2217*x+0.0046	0.9998	0.1-5.0	0.042	0.00	94.37 (85.47-104.70)
Ti (μg/L)	Y=3.7724E-004*x+1.0540E-004	0.9999	0.5-100	0.55	73.45	6.96 (0.39-3.18)
V (μg/L)	Y=0.1448*x+0.0013	1	0.1-100	0.015	14.55	0.26 (0.11-0.30)
Cr (μg/L)	Y=0.1910*x+0.0114	1	0.1-100	0.078	16.73	4.08 (0.63-4.48)
Mn (μg/L)	Y=0.0956*x+0.0043	0.9999	0.1-100	0.057	18.91	4.02 (0.70-4.06)
Fe (μg/L)	Y=0.1606*x+0.1456	1	10-500	1.88	0.00	1289.47 (951.10-1537.47)
Co (μg/L)	Y=0.2800*x+0.0038	0.9999	0.1-100	0.017	1.09	0.80 (0.30-0.72)
Ni (μg/L)	Y=0.0103*x+0.0033	0.9993	0.5-100	0.17	18.55	18.98 (1.59-24.87)
Cu (μg/L)	Y=0.0299*x+0.0309	0.9997	0.5-100	1.1	0.00	9983.43 (8187.41-11063.98)
Zn (μg/L)	Y=0.0244*x+0.0569	0.9999	0.5-100	1.34	0.00	974.25 (803.15-1117.72)
Se (μg/L)	Y=0.0016*x+4.2796E-004	0.9995	0.5-100	1.08	0.36	118.86 (98.84-140.28)
Mo (μg/L)	Y=0.1298*x+0.0020	1	0.1-100	0.032	0.36	1.22 (0.86-1.45)
Ag (μg/L)	Y=0.5571*x+0.0013	1	0.1-100	0.007	20.36	0.11 (0.02-0.12)
Cd (μg/L)	Y=0.0699*x+5.9152E-005	1	0.1-100	0.008	50.91	0.10 (0.01-0.06)
Sb (μg/L)	Y=0.3580*x+0.0020	1	0.1-100	0.004	0.36	2.55 (2.04-2.92)
Ba (μg/L)	Y=0.1057*x+0.0103	0.9998	0.5-100	0.035	34.18	3.51 (0.02-4.18)
Tl (μg/L)	Y=2.3409*x+8.7091E-004	1	0.1-100	0.004	37.09	0.07 (0.00-0.04)
Pb (μg/L)	Y=1.5111*x+0.0997	1	0.1-100	0.008	42.91	1.15 (0.01-1.15)

Abbreviations: LOD, limit of dection; Be, beryllium; Na, sodium; Mg, magnesium; K, potassium; Ca, calcium; Ti, titanium; V, vanadium; Cr, chromium; Mn, manganese; Fe, iron; Co, cobalt; Ni, nickel; Cu, copper; Zn, zinc; Se, selenium; Mo, molybdenum; Ag, silver; Cd, cadmium; Sb, antimony; Ba, barium; Tl, thallium; Pb, lead.

Supplementary Table S2. Spearman correlations among serum concentrations of 19 elements in control group and uRM group.

group		Na	Mg	K	Ca	V	Cr	Mn	Fe	Co	Ni	Cu	Zn	Se	Mo	Ag	Sb	Ba	Tl	Pb
control	Na	1.000	0.368*	0.138	0.282	0.268	0.157	0.325*	0.061	0.193	0.100	0.343*	0.134	0.202	0.262	0.483**	0.174	-0.282	-0.038	-0.191
uRM		1.000	0.123	-0.021	0.064	0.221**	0.207**	0.222**	0.141*	-0.123	-0.175**	0.413**	-0.040	0.212**	0.250**	0.190**	0.174**	-0.308**	0.120	-0.257**
control	Mg	0.368*	1.000	0.466**	0.590**	0.168	-0.074	-0.122	0.221	0.269	0.042	0.331*	0.338*	0.389**	0.109	0.191	0.185	0.043	0.202	-0.005
uRM		0.123	1.000	0.667**	0.767**	0.102	0.128	0.115	0.280**	0.215**	0.373**	0.170**	0.422**	0.320**	0.148*	-0.059	0.266**	0.276**	0.086	0.186**
control	K	0.138	0.466**	1.000	0.631**	0.099	-0.144	-0.015	0.344*	0.232	-0.138	0.319*	0.496**	0.582**	0.151	0.077	0.213	0.132	0.469**	-0.053
uRM		-0.021	0.667**	1.000	0.550**	0.043	0.098	0.037	0.456**	0.098	0.167*	0.119	0.493**	0.264**	0.081	-0.116	0.107	0.197**	0.061	0.040
control	Ca	0.282	0.590**	0.631**	1.000	0.009	-0.021	-0.245	0.157	0.341*	0.149	0.088	0.338*	0.522**	0.447**	0.093	0.365*	0.182	0.325*	-0.113
uRM		0.064	0.767**	0.550**	1.000	0.149*	0.159*	0.188**	0.172**	0.222**	0.468**	0.182**	0.398**	0.493**	0.176**	0.038	0.278**	0.341**	-0.017	0.176**
control	V	0.268	0.168	0.099	0.009	1.000	0.377*	0.604**	0.082	0.024	-0.010	0.078	0.287	0.086	-0.035	0.250	0.035	0.022	0.312*	0.214
uRM		0.221**	0.102	0.043	0.149*	1.000	0.588**	0.758**	0.009	0.234**	0.242**	0.140*	0.228**	0.430**	0.347**	0.579**	0.302**	0.027	0.283**	0.155*
control	Cr	0.157	-0.074	-0.144	-0.021	0.377*	1.000	0.226	-0.042	0.079	0.026	-0.370*	-0.048	0.138	0.044	0.223	-0.074	0.173	-0.211	0.003
uRM		0.207**	0.128	0.098	0.159*	0.588**	1.000	0.729**	0.092	0.338**	0.308**	0.149*	0.159*	0.255**	0.193**	0.189**	0.077	0.154*	-0.035	0.213**
control	Mn	0.325*	-0.122	-0.015	-0.245	0.604**	0.226	1.000	0.090	0.229	0.126	0.120	0.213	-0.055	-0.064	0.291	-0.015	0.080	0.192	0.329*
uRM		0.222**	0.115	0.037	0.188**	0.758**	0.729**	1.000	0.079	0.284**	0.306**	0.218**	0.212**	0.395**	0.265**	0.369**	0.270**	0.034	0.197**	0.195**
control	Fe	0.061	0.221	0.344*	0.157	0.082	-0.042	0.090	1.000	-0.145	0.195	0.275	0.073	0.287	-0.035	0.067	-0.228	0.041	0.348*	-0.134
uRM		0.141*	0.280**	0.456**	0.172**	0.009	0.092	0.079	1.000	0.028	-0.037	0.092	0.349**	0.137*	-0.069	-0.056	0.082	0.011	-0.009	-0.076

control	Co	0.193	0.269	0.232	0.341*	0.024	0.079	0.229	-0.145	1.000	0.065	-0.111	0.151	0.299	-0.047	0.168	0.513**	0.055	0.229	-0.033
uRM		-0.123	0.215**	0.098	0.222**	0.234**	0.338**	0.284**	0.028	1.000	0.414**	-0.074	0.230**	0.124	0.075	0.058	0.069	0.303**	0.074	0.434**
control	Ni	0.100	0.042	-0.138	0.149	-0.010	0.026	0.126	0.195	0.065	1.000	0.045	-0.036	-0.144	0.259	-0.063	-0.088	0.114	0.099	0.085
uRM		-0.175**	0.373**	0.167*	0.468**	0.242**	0.308**	0.306**	-0.037	0.414**	1.000	-0.144*	0.366**	0.100	0.087	0.109	0.115	0.537**	-0.112	0.515**
control	Cu	0.343*	0.331*	0.319*	0.088	0.078	-0.370*	0.120	0.275	-0.111	0.045	1.000	0.236	-0.017	-0.027	0.138	0.027	-0.074	0.125	0.106
uRM		0.413**	0.170**	0.119	0.182**	0.140*	0.149*	0.218**	0.092	-0.074	-0.144*	1.000	0.021	0.246**	0.111	0.052	0.130*	-0.230**	0.082	-0.190**
control	Zn	0.134	0.338*	0.496**	0.338*	0.287	-0.048	0.213	0.073	0.151	-0.036	0.236	1.000	0.217	0.052	-0.112	0.031	0.307*	0.195	0.235
uRM		-0.040	0.422**	0.493**	0.398**	0.228**	0.159*	0.212**	0.349**	0.230**	0.366**	0.021	1.000	0.226**	0.001	0.048	0.254**	0.409**	0.130*	0.341**
control	Se	0.202	0.389**	0.582**	0.522**	0.086	0.138	-0.055	0.287	0.299	-0.144	-0.017	0.217	1.000	0.076	0.412**	0.396**	-0.099	0.368*	-0.254
uRM		0.212**	0.320**	0.264**	0.493**	0.430**	0.255**	0.395**	0.137*	0.124	0.100	0.246**	0.226**	1.000	0.171**	0.430**	0.423**	-0.099	0.324**	-0.133*
control	Mo	0.262	0.109	0.151	0.447**	-0.035	0.044	-0.064	-0.035	-0.047	0.259	-0.027	0.052	0.076	1.000	0.191	0.099	-0.040	-0.014	0.064
uRM		0.250**	0.148*	0.081	0.176**	0.347**	0.193**	0.265**	-0.069	0.075	0.087	0.111	0.001	0.171**	1.000	0.250**	0.114	0.047	0.145*	0.002
control	Ag	0.483**	0.191	0.077	0.093	0.250	0.223	0.291	0.067	0.168	-0.063	0.138	-0.112	0.412**	0.191	1.000	0.265	-0.143	0.006	0.047
uRM		0.190**	-0.059	-0.116	0.038	0.579**	0.189**	0.369**	-0.056	0.058	0.109	0.052	0.048	0.430**	0.250**	1.000	0.279**	-0.082	0.354**	0.058
control	Sb	0.174	0.185	0.213	0.365*	0.035	-0.074	-0.015	-0.228	0.513**	-0.088	0.027	0.031	0.396**	0.099	0.265	1.000	-0.148	0.438**	-0.027
uRM		0.174**	0.266**	0.107	0.278**	0.302**	0.077	0.270**	0.082	0.069	0.115	0.130*	0.254**	0.423**	0.114	0.279**	1.000	-0.057	0.396**	-0.056
control	Ba	-0.282	0.043	0.132	0.182	0.022	0.173	0.080	0.041	0.055	0.114	-0.074	0.307*	-0.099	-0.040	-0.143	-0.148	1.000	-0.223	0.386*

uRM		-0.308**	0.276**	0.197**	0.341**	0.027	0.154*	0.034	0.011	0.303**	0.537**	-0.230**	0.409**	-0.099	0.047	-0.082	-0.057	1.000	-0.242**	0.550**
control	Tl	-0.038	0.202	0.469**	0.325*	0.312*	-0.211	0.192	0.348*	0.229	0.099	0.125	0.195	0.368*	-0.014	0.006	0.438**	-0.223	1.000	0.033
uRM		0.120	0.086	0.061	-0.017	0.283**	-0.035	0.197**	-0.009	0.074	-0.112	0.082	0.130*	0.324**	0.145*	0.354**	0.396**	-0.242**	1.000	-0.023
control	Pb	-0.191	-0.005	-0.053	-0.113	0.214	0.003	0.329*	-0.134	-0.033	0.085	0.106	0.235	-0.254	0.064	0.047	-0.027	0.386*	0.033	1.000
uRM		-0.257**	0.186**	0.040	0.176**	0.155*	0.213**	0.195**	-0.076	0.434**	0.515**	-0.190**	0.341**	-0.133*	0.002	0.058	-0.056	0.550**	-0.023	1.000

Abbreviation: Be, beryllium; Na, sodium; Mg, magnesium; K, potassium; Ca, calcium; Ti, titanium; V, vanadium; Cr, chromium; Mn, manganese; Fe, iron; Co, cobalt; Ni, nickel; Cu, copper; Zn, zinc; Se, selenium; Mo, molybdenum; Ag, silver; Cd, cadmium; Sb, antimony; Ba, barium; Tl, thallium; Pb, lead.
Note: correlations among different metals were conducted by Spearman analysis. *Correlation is significant at the 00.05 level (2-tailed); **Correlation is significant at the 00.01 level (2-tailed)

Supplementary Table S3. The association of uRM with the WQS index of metals.

Model	Direction	index	Estimate	Std. Error	z value	*P*
Crude	Positive	WQS	1.300	0.611	2.120	0.034
	Negative	WQS	-1.420	0.445	-3.200	0.001
Adjusted	Positive	WQS	1.560	0.655	2.380	0.017
		age	0.076	0.058	1.310	0.189
		BMI	-0.082	0.095	-0.861	0.389
		Education level	-0.464	0.349	-1.330	0.183
	Negative	WQS	-1.460	0.468	-3.120	0.002
		age	0.067	0.058	1.160	0.247
		BMI	-0.050	0.098	-0.519	0.604
		Education level	-0.576	0.350	-1.640	0.101

Supplementary Table S4. Association between metal composite levels and uRM based on weighted quantile sum (WQS) regression analysis.

	WQS index	mean_weight	mean_weight (adjusted*)
Mg	Positive	0.000	0.000
	Negative	0.054	0.048
Ca	Positive	0.000	0.000
	Negative	0.499	0.391
V	Positive	0.025	0.060
	Negative	0.015	0.005
Cr	Positive	0.020	0.011
	Negative	0.019	0.023
Mn	Positive	0.008	0.004
	Negative	0.009	0.016
Fe	Positive	0.087	0.065
	Negative	0.013	0.008
Co	Positive	0.006	0.011
	Negative	0.015	0.028
Ni	Positive	0.267	0.240
	Negative	0.000	0.000
Cu	Positive	0.012	0.004
	Negative	0.056	0.031
Zn	Positive	0.054	0.091
	Negative	0.000	0.000
Se	Positive	0.000	0.000
	Negative	0.144	0.233
Mo	Positive	0.028	0.015
	Negative	0.043	0.033
Ag	Positive	0.035	0.015
	Negative	0.032	0.084
Sb	Positive	0.071	0.028
	Negative	0.000	0.001
Ba	Positive	0.052	0.070
	Negative	0.006	0.004
Tl	Positive	0.031	0.041
	Negative	0.095	0.094
Pb	Positive	0.304	0.345
	Negative	0.000	0.000

Abbreviation: Mg, magnesium; Ca, calcium; V, vanadium; Cr, chromium; Mn, manganese; Fe, iron; Co, cobalt; Ni, nickel; Cu, copper; Zn, zinc; Se, selenium; Mo, molybdenum; Ag, silver; Sb, antimony; Ba, barium; Tl, thallium; Pb, lead. *Adjusted by age BMI and education level.
